# Improving prediction of blood cancer using leukemia microarray gene data and Chi2 features with weighted convolutional neural network

**DOI:** 10.1038/s41598-024-65315-7

**Published:** 2024-07-07

**Authors:** Ebtisam Abdullah Alabdulqader, Aisha Ahmed Alarfaj, Muhammad Umer, Ala’ Abdulmajid Eshmawi, Shtwai Alsubai, Tai-hoon Kim, Imran Ashraf

**Affiliations:** 1 Department of Information Technology, College of Computer and Information Sciences, King Saud University, P. O. Box 800, 11421 Riyadh, Saudi Arabia; 2 Department of Information Systems, College of Computer and Information Sciences, Princess Nourah bint Abdulrahman University, P.O. Box 84428, 11671 Riyadh, Saudi Arabia; 3 Department of Computer Science and Information Technology, The Islamia University of Bahawalpur, Bahawalpur, 63100 Pakistan; 4 Department of Cybersecurity, College of Computer Science and Engineering, University of Jeddah, Jeddah, 23218 Saudi Arabia; 5 Department of Computer Science, College of Computer Engineering and Sciences, Prince Sattam bin Abdulaziz University, P.O. Box 151, 11942 Al-Kharj, Saudi Arabia; 6 School of Electrical and Computer Engineering, Yeosu Campus, Chonnam National University, 50, Daehak-ro, Yeosu-si, 59626 Jeollanam-do Republic of Korea; 7 Department of Information and Communication Engineering, Yeungnam University, Gyeongsan, 38541 Korea

**Keywords:** Computational biology and bioinformatics, Health care

## Abstract

Blood cancer has emerged as a growing concern over the past decade, necessitating early diagnosis for timely and effective treatment. The present diagnostic method, which involves a battery of tests and medical experts, is costly and time-consuming. For this reason, it is crucial to establish an automated diagnostic system for accurate predictions. A particular field of focus in medical research is the use of machine learning and leukemia microarray gene data for blood cancer diagnosis. Even with a great deal of research, more improvements are needed to reach the appropriate levels of accuracy and efficacy. This work presents a supervised machine-learning algorithm for blood cancer prediction. This work makes use of the 22,283-gene leukemia microarray gene data. Chi-squared (Chi2) feature selection methods and the synthetic minority oversampling technique (SMOTE)-Tomek resampling is used to overcome issues with imbalanced and high-dimensional datasets. To balance the dataset for each target class, SMOTE-Tomek creates synthetic data, and Chi2 chooses the most important features to train the learning models from 22,283 genes. A novel weighted convolutional neural network (CNN) model is proposed for classification, utilizing the support of three separate CNN models. To determine the importance of the proposed approach, extensive experiments are carried out on the datasets, including a performance comparison with the most advanced techniques. Weighted CNN demonstrates superior performance over other models when coupled with SMOTE-Tomek and Chi2 techniques, achieving a remarkable 99.9% accuracy. Results from k-fold cross-validation further affirm the supremacy of the proposed model.

## Introduction

Cancer has become a leading cause of death globally. It happens because of different problems in the body or, diseases passed down in families^1–3^. The World Health Organization (WHO) reports that cancer is the contributing factor of mortality for people below 70 years old in 112 countries out of 183. In 23 or more countries, cancer ranks as the third or fourth leading cause of death^4^. There are not many effective treatments for the most common types of cancer. Usually, doctors check for cancer by looking at tiny bits of tissue under a microscope^5^. But this way of cancer diagnosis is expensive, takes a lot of time, and can sometimes lead to wrong diagnosis.

Cancer occurs when abnormal cells proliferate uncontrollably within the body and have the potential to spread. In 2020, there were 19.3 million new cases of cancer, resulting in nearly 10 million deaths from the disease. Survival probabilities vary for each type of cancer. For example, the death rate from lung cancer was 18%, the death rate from colon cancer was 9.4%, and the rates for liver, breast, and stomach cancer were 7.7%, and 6.9%,8.3%, respectively. Blood cancers accounted for approximately 10% of newly diagnosed cancer cases^6^.

Microarray analysis methods can be categorized into five groups: (i) examining differently expressed genes, (ii) analyzing pairs of differentially expressed genes, (iii) elucidating gene functions, (iv) grouping genes based on similarities, and (v) employing classification analysis. In this study, our emphasis was on classification analysis, a widely utilized statistical approach to deduce or predict the category in which certain data belongs. This method finds frequent application in cancer research.

Leukemia, a form of blood cancer, shares classification with Lymphoma and Myeloma. In leukemia, excessive production of immature white blood cells occurs, leading to the overcrowding of the bone marrow and hindering the generation of other vital blood cells crucial for a robust immune system and overall blood health^7^. Pathologists, specializing in leukemia diagnosis, employ various techniques, including Array-based Comparative Genomic Hybridization, Long-Distance Inverse Polymerase Chain Reaction (LDI-PCR), and Molecular Cytogenetics (ACGH)^8^. These methodologies demand considerable expertise and effort^9^. Medical practitioners also conduct blood and bone marrow tests to detect leukemia, albeit these procedures are both costly and time-consuming^8,10,11^. Interventional radiology, another approach, has limitations due to familial traits influencing the imaging^8^. Presently, deep learning (DL) methods play a vital role in aiding medical diagnoses, particularly in identifying leukemia cells.

Analyzing and categorizing gene expression using DNA microarray data is a valuable method for diagnosing and predicting specific types of cancer. Artificial intelligence (AI) tools, such as algorithms, play a crucial role in understanding gene expression data and classifying genes, particularly in the context of cancer. This article will explore different strategies found in the literature, examine the datasets used, and assess their advantages and disadvantages. In the field of medical research on cancer, traditional deep learning techniques like autoencoders, artificial neural networks (ANN), and convolutional neural networks (CNN) are essential. They help in decision-making related to the diagnosis and treatment of diseases like cancer. As time passes, diseases in general, and especially cancer, have become more complex, making them harder to understand, analyze, and treat. Cancer research is a significant and intricate topic in the field of medicine^12^. Given the significance of utilizing gene data for blood cancer detection, this study contributes in the following ways:

- To enhance the predictive accuracy of blood cancer detection, a novel hybrid model weighted CNN is introduced. The weighted CNN combines three CNN models through a majority voting mechanism. The impact of the synthetic minority oversampling technique (SMOTE)-Tomek on data balancing is explored. hi-square (Chi2) is used to determine the best set of features for classification, and SMOTE-Tomek (synthetic minority oversampling technique) is used to equalize the class-imbalance problem using the upsampling technique.
- The performance of well-known machine learning methods on microarray gene data is evaluated in this work. These techniques include decision trees (DT), AdaBoost classifier (ADA), k-nearest neighbor (KNN), random forest (RF), logistic regression (LR), support vector classifier (SVC), and Naive Bayes (NB).
- The effectiveness of the proposed approach is thoroughly examined through extensive experiments, and a comparative analysis with various state-of-the-art methods is conducted. K-fold cross-validation is used to further substantiate the results in order to validate the robustness of the proposed approach.

The rest of the paper follows a structured format. Section delves into the latest advancements in DNA microarray techniques, providing a detailed examination of specific methodologies. Moving on to “Material and methods”, we present the proposed approach and elucidate the microarray datasets utilized in our experiments. “Experiments and analysis” outlines the outcomes of these experiments. It wraps up the study in “Conclusion”, where we provide a summary of the findings and explore possible directions for further research.

## Related work

Using microarray data to classify cancer is a common research area in machine learning. Researchers propose and evaluate various methods and models for this task. Karim et al^13^ proposed an automated system for leukemia cancer classification using the microarray gene data. They used machine learning and ensemble learning models for the prediction of leukemia. To handle the class imbalance problem they used the SMOTE upsampling method. The result of the study shows that the proposed LDSVM achieved an accuracy score of 99.8%. Similar to our work^14^, another study examined the diagnostic performance of a deep learning approach using the Leukemia_GSE9476 dataset in comparison to conventional methods. The dataset had gene data from Leukemia microarrays, including 22,283 genes. They used normalization tests at the beginning, and for training and testing, they used a DNN neural network. The results showed that the accuracy of the deep learning network was 0.96%, while the accuracy of the standard technique was 0.63%.

The Leukemia_GSE28497 dataset was utilized in a different study^15^ to assess the interoperability of several microarray and ribonucleic acid (RNA)-seq systems. In their investigation, they examined four different kinds of leukemia samples. To choose the best features, they used a method called minimum redundancy maximum relevance (mRMR). Findings indicate that a small fraction of ten genes can yield an accuracy of 97.29% . To confirm the model’s effectiveness for multi-class classification, the statistical test known as analysis of variance (ANOVA) is run. For the multi-class classification Fauzi et al.^16^ proposed an automated system that uses the Fuzzy Support Vector Machine with the feature selection technique PCA. They performed the experiments in two scenarios. In the first experiment, they used the FSVM on the original features, and in the second experiment, they used the FSVM on the PCA features. The result of the study shows that the proposed FSVM outperformed the PCA features and achieved an accuracy score of 96.92%, whereas on the original features it achieved an accuracy score of 87.69%.

Abd El-Nasser et al.^17^developed an Enhanced Classification Algorithm (ECA) that combines the Select Most Informative Genes (SMIG) module with standardization, achieving 98% accuracy in just 0.1 seconds when preprocessing and classification are performed. This approach outperformed previous methods. In a separate study, Sanaz Mehrabani et al.^18^ employed machine learning models and sparsity-based gene selection techniques to detect blood cancer using leukemia gene expression data. They employed two machine learning models such as RF and SVM for the prediction of blood cancer. Results of the study reveal that the RF achieved the highest accuracy on the ten genes selected by norm and norm sparsity-based gene selection and SVM achieved the highest accuracy on the norm method. Mahdi et al^19^ proposed a fusion of the stochastic gradient descent approach with supervised principal component analysis (SPCA) for the leukemia prognosis. This system has the ability to handle the large dimensional space efficiently. The results reveal that SGD-SPCA stands out as a highly efficient method, achieving an impressive 99.1% accuracy on the training data. In a separate study, Loey et al.^20^ developed classification models to distinguish between blood microscopic images of individuals with and without leukemia. They leveraged a pre-trained Convolutional Neural Network (CNN) called AlexNet for feature extraction, combined with various classifiers. The results show that Support Vector Machine (SVM) outperformed other classifiers in terms of performance. Moreover, AlexNet demonstrated its superiority over other models across different metrics when used for both feature extraction and classification.

In reference to cancer prediction, a multi-model ensemble is introduced by Yawen Xiao et al.^21^. This work examined gene data that were taken from lung, breast, and stomach tissues. To prevent overfitting in classification, the authors employed the DESeq approach, which effectively pinpointed genetic distinctions between normal and tumor phenotypes. Additionally, this approach managed data dimensionality, leading to improved forecast accuracy and a noteworthy reduction in computational time. They achieved the highest accuracy of 98.78% using a deep learning ensemble model. Ancona et al.^22^ explore gene tumors related to colon cancer in their study. Their results highlight that the voting categories yield optimal outcomes when applied to an individual classifier. Cancer classification involves constructing models on datasets, specifically those containing microarray gene expression information. The process involves discerning the class value for each case in the sample by creating the model. Diagnostic findings based on this approach may empower doctors to implement a suitable therapy protocol, especially during the early stages of diagnosis and treatment.

Despite the impressive outcomes detailed in the aforementioned studies, the utilization of microarray gene data for predicting blood cancer remains relatively underexplored. Furthermore, aside from a few research endeavors, the reported accuracy in the remaining studies is insufficient for effective blood cancer prediction. The complete summary of the related work is shown in Table 1.

**Table 1:** Summary of the related work.

Ref	Classifiers used	Dataset	Achieved accuracy
^13^	LR, NB, RF, GBM, Linear regression, SVM, LDSVM	NCBI/GEO public database	99.8% LDSVM
^14^	DNN	NCBI/GEO public database	96.6% DNN
^15^	RF, SVM, NB, and KNN	NCBI/GEO public database	NB: 97.29%,
^16^	Fuzzy SVM with PCA	http://www.gems-system.org/	96.92% FSVM
^17^	NB updateable, NB Tree, Conjunction Rule, BNL, Random Subspace, PART, and ECA	Three datasets contains 7130 Genes	97.22% ECA
^18^	SVM, RF	7129 genes Microarray dataset	RF 100% SVM 100%
^19^	NB, KNN, DT, SGD-PCA	SRBCT dataset	99.1% SGD-SPCA
^20^	AlexNet	2,820 images	100%
^21^	RF, GBDT, KNN, SVM, DT, Majority voting, Deep learning ensemble model	Three RNA-seq data sets	98.78% deep learning ensemble model
^22^	Weighted Voting Algorithm (WVA), Regularized Least Squares (RLS) and Support Vector Machine (SVM)	Colorectal cancer(CRC) at CSS hospital dataset	SVM, error rate of e = 11%

## Material and methods

**Figure 1:**
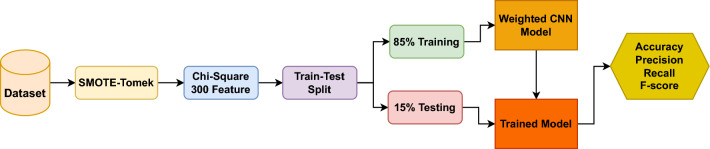
Proposed methodology diagram.

This section contains details on the dataset, techniques, and methods applied to blood cancer prediction. Step by step complete proposed methodology diagram is shown in Fig. 1.

### Description of dataset

This study uses the Leukemia_GSE28497 dataset^23^. There are seven classifications, 22,283 genes (features), and 281 samples in total. Class-wise count of the dataset is given in Table 2.

**Table 2:** Class-wise count of the dataset.

Target	Count
B-CELL-ALL	74
B-CELL-ALL-HYPERDIP	51
B-CELL-ALL-TCF3-PBX1	22
B-CELL-ALL-MLL	17
B-CELL-ALL-T-ALL	46
B-CELL-ALL-ETV6-RUNX1	53
B-CELL-ALL-HYPO	18
TOTAL SAMPLES	281

In Table 3, the original dataset example is provided. This explains the real data that was obtained by applying the microarray gene technique. Blood cancer types are described by attributes, while the properties of the genes that are utilized to distinguish affected individuals from unaffected individuals are shown in another column. A microarray test is used to determine the values of these features. Every instance is described in a single row and contains information about the blood sample monitored by the microarray.

**Table 3:** Sample from the dataset.

Type	BCELL_ALL	BCELL_ALL
1053_at	5.009216	5.415108
1007_s_at	7.409521	7.177109
AFFXTrpnXM_at	2.608381	2.634063

### Data preprocessing

The dataset was made available via the National Centre for Biotechnology Information (NCBI) website. Subsequently, preprocessing steps are implemented to enhance the effectiveness of learning models^24^. This includes data resampling for balancing the dataset using the SMOTE-Tomek technique, and generating data for minority classes, as detailed in Table 4.

**Table 4:** Count of the target after SMOTE-Tomek.

Target	Count	After SMOTE-Tomek
B-CELL-ALL	74	74
B-CELL-ALL-TCF3-PBX1	22	73
B-CELL-ALL-HYPERDIP	51	74
B-CELL-ALL-HYPO	18	74
B-CELL-ALL-MLL	17	76
B-CELL-ALL-T-ALL	46	74
B-CELL-ALL-ETV6-RUNX1	53	75
TOTAL SAMPLES	281	520

The Chi-square method is then used for feature selection in order to reduce complexity. The top 300 gene features for the best learning model fitting are given in Table 5.

**Table 5:** Count of features after chi-2 feature extraction technique.

Features	Count
Original	22,283
After Chi-square	300

The selection of features is guided by empirical findings. The preprocessed dataset is then divided in an 85:15 ratio into training and testing sets given in Table 6.

**Table 6:** Training testing ratio of all features.

Techniques	Training set		Testing set	
	Sample	Features	Sample	Features
Original dataset	238	22,283	43	22,283
SMOTE-Tomek	432	22,283	77	22,283
Chi2	238	300	43	300
SMOTE-Tomek +Chi2	432	300	77	300

With 85% dedicated to training, the models receive ample data for effective learning, given the dataset’s overall size. Post-training, the remaining 15% is utilized to assess the model’s performance using measures like F1 score, recall, accuracy, and precision. This preprocessing ensures the dataset is well-prepared for training and evaluation, enhancing the learning models’ performance.

### SMOTE-Tomek

SMOTE-Tomek is a technique that addresses the issue of overlap by combining SMOTE and Tomek Links. Tomek Links identifies pairs of instances from different classes and introduces new instances randomly^25^. If the distance between the new instance and either selected pair is smaller than the distance of the pair itself, the chosen pair is eliminated. In essence, Tomek Links can eliminate instances situated at the boundaries of multiple classes, considering the adjacencies between instances near these boundaries. It targets instances considered to belong to multiple classes due to SMOTE, thereby eliminating those responsible for overlap. However, in this scenario, the instance being removed is not likely to be adjacent to its proper class, potentially increasing the chance of introducing unwanted noise.

### Chi-square

Chi-square is a non-parametric statistical method used for feature selection, specifically selecting the top n features. It is widely employed in data analysis tasks. Chi-2 assesses the independence between a given phrase and the presence of a specific class. In a given document D, the score for each term is estimated and ranked using the Eq. (1)

1 $$\begin{aligned} X^2=(D,t,c)=\sum _{e_t\varepsilon \left\{ 0,1\right\} } \sum _{e_c\varepsilon \left\{ 0,1\right\} } \frac{(Ne_t e_c)^2}{Ee_t e_c} \end{aligned}$$

In this context, let *N* represent the observed frequency and *E* denote the predicted frequency. When *t* is present, the variable $4_t$ is assigned a value of 1; otherwise, it is assigned a value of 0, and $e_c$ is allocated a value of 1 if the document is part of the *c* class and 0 otherwise. The null hypothesis *H*0, which asserts independence (i.e., that the document class does not influence the term’s frequency), should be rejected, according to a significant Chi2 score for each characteristic. This implies that there is interdependence between the feature and the class. Consequently, in such instances, it is advisable to select the microarray gene feature for model training.

### Supervised machine learning models

This study uses a variety of machine learning models, such as KNN, RF, LR, ETC, SVC, ADA, NB, DT, and the proposed approach, WVCNN, to predict blood cancer. Table 7 provides implementation details about these machine learning models and their hyperparameter settings for all of them. To find the best parameters, a method called grid search is used. This involves trying different values for each parameter within a specified range and evaluating how well the model works. Every parameter goes through the procedure, and the values that optimize the model’s performance are selected at the conclusion.

**Table 7:** Hyper-parameter tuning of all supervised learning models.

Classifier	Hyperparameter setting
RF	random_state = 2, max_depth = 60, n_estimators = 300
LR	solver = ‘saga’, C D 3:0, max_iter = 100, penalty = ‘l2’
ADA	n_estimators = 300, learning_rate = 0.2, max_depth = 300
DT	max_depth = 25, max_depth = 25
KNN	n_neighbors = 4, n_neighbors = 6
ETC	n_estimators = 300, random_state = 5, max_depth = 300
NB	Default setting
SVC	kernel = ‘linear’, C D 2:0, random_state = 500

#### Random forest

RF is a model that predicts very accurately by using a group of decision trees^26^. It combines the results from many decision trees. This model uses a technique called bagging, where it trains various decision trees on different groups of data. In each group, the training data is sampled with replacement, which means some data might be repeated. The size of this sample is like the size of the original training data. When making predictions, RF and other classifiers follow similar processes for creating decision-making groups. A key difficulty in developing these models is deciding the attributes of the main decision point at each step, determined using Eq. (2).

2 $$\begin{aligned} F(x_t)= \frac{1}{B}\sum _{i=0}^{B} F_i (x_t) \end{aligned}$$

#### Logistic regression

LR is a method in mathematics that processes information using one or more variables to find a solution^27^. It’s specifically used for predicting probabilities of class membership when dealing with categorical target variables. LR uses a logistic function to assess the likelihood of a relationship between the dependent variable and one or more independent variables. It is considered the most suitable learning model when dealing with categorical target variables. Equation (3) shows the logistic function used by LR

3 $$\begin{aligned} f(x)=\frac{L}{1+ e^{-m(v-v_0)}} \end{aligned}$$

#### Support vector classifier

Classification involves organizing a dataset into categories using specific criteria to provide a more meaningful classification^28^. The classification approach known as SVC is based on the support vector methodology. SVC’s primary goal is to identify the best-fitting hyperplane that effectively divides or arranges the given data. You may enter characteristics into the classifier to ascertain the anticipated class after the hyperplane has been constructed. This algorithm is well-suited for various applications, including our purpose, as it can be employed in different contexts.

#### K-Nearest neighbors

KNN is a fundamental machine learning model that is used for problems involving both regression and classification^29,30^. This model assigns a data point to a class based on its closest neighbors, determining proximity through a distance attribute. In this experiment, the effectiveness of KNN is demonstrated, particularly when k is set to five (k = 5). This signifies that the model considers the five nearest neighbors and selects a class according to the closest or majority distance.

#### Naive Bayes

The NB algorithm is an algorithm for classification problems that focuses on the Bayes theorem^31^. It is a supervised learning algorithm known for its efficiency and scalability in training with a restricted set of information. As a probabilistic classifier, the likelihood that an item will belong to a specific class is predicted by NB. One key assumption of the NB classifier is that each feature’s likelihood is independent of others, and they don’t overlap. This suggests that every attribute has an equal role in identifying whether a sample is a member of a certain class. The NB classifier is simple to use, computes rapidly, and performs well on large, highly dimensional datasets.

#### Extra trees classifier

The ETC operates similarly to the random forest, with the main difference lying in the tree-building process^32^. Every decision tree in ETC is built with the first training sample. The Gini index is used to find the optimum way to split the data inside the tree, and k samples from the best solutions are used to make judgments. This results in the creation of multiple decision trees Using these examples of random function indicators, aiming to ensure they are not highly correlated. The decision tree algorithm is versatile, suitable for both categorical and numerical data, and it performs effectively in various scenarios.

#### Decision tree

The DT is a widely used and potent data-mining technique that has been extensively developed and tested by numerous researchers^33^. Despite its effectiveness, it is essential to acknowledge the presence of data errors during the learning process. Consequently, working with substantial volumes of data is crucial to creating a decision tree algorithm capable of producing a straightforward tree structure with high classification accuracy. For this study, a dataset from Kaggle was chosen to implement the decision tree algorithms. The strategic division of data significantly impacts the accuracy of the tree, with different decision criteria utilized for classification and regression tasks.

The entropy is expressed mathematically for 1 attribute by Eq. (4)

4 $$\begin{aligned} Entropy(S)= \sum _{i=1}^{c} - P_ilog_2P_i \end{aligned}$$

Entropy is expressed mathematically for multiple attributes by Eq. (5)

5 $$\begin{aligned} Entropy(T,X) = \sum _{c \varepsilon X} P(c) E (c) \end{aligned}$$

IG is defined mathematically by Eq. (6)

6 $$\begin{aligned} Information Gain (T,x) =Entropy (T) - Entropy (T, X) \end{aligned}$$

#### AdaBoost

This group learning model uses a method called boosting to teach unskilled students, like decision trees. ADA, short for adaptive boosting, is important in history^34^. It was the first system that could change and help weak learners. The ADA method brings together ’weak learners,’ teaching them one after the other back on copies of the first data set. All weaker learners concentrate on tough data points or unusual numbers. It works like an overall model, using N repeats of weak learners trained on the same set of features. These are given different weights. ADA shows it works with strong math reasons. Many experiments have shown that ADA, a type of machine learning system, is often better than other systems.

The study used the ADA method with different values. It adjusted these values well to get high accuracy. The ’n_estimator’ value was set to 300. This means that the ADA machine used 300 little learners to make guesses. It’s important to point out the difference between RF and ADA. RF uses a method called bagging, while ADA uses a boosting strategy. Boosting should be understood as combining weak learners into one strong learner by putting weight on them in a specific way. ’Random_state’ is another factor used. It controls how random the samples are during training for predicting models.

#### Artificial neural networks

Artificial neural networks (ANNs) are computational models inspired by the human brain’s neural networks, designed to recognize patterns and solve complex problems^35^. ANNs consist of interconnected layers of neurons, including input, hidden, and output layers, where each neuron processes input data and passes it through an activation function. These models are widely used in various applications such as image and speech recognition, natural language processing, and predictive analytics due to their ability to learn and generalize from data. Training an ANN involves adjusting the weights of the connections between neurons using algorithms like backpropagation to minimize error. Despite their power, ANNs can require large amounts of data and computational resources to achieve high performance.

#### Multi-layer perceptron

Multi-layer perceptron (MLP) is a type of artificial neural network that consists of multiple layers of neurons, typically including an input layer, one or more hidden layers, and an output layer^30^. Each neuron in an MLP uses a nonlinear activation function to process input data, allowing the network to capture complex patterns and relationships in the data. MLPs are particularly effective for supervised learning tasks such as classification and regression. The backpropagation algorithm is commonly used to train MLPs by adjusting the weights of the connections to minimize the error between the predicted and actual outputs. MLPs have been successfully applied in various fields, including image and speech recognition, finance, and healthcare.

### Proposed methodology

**Figure 2:**
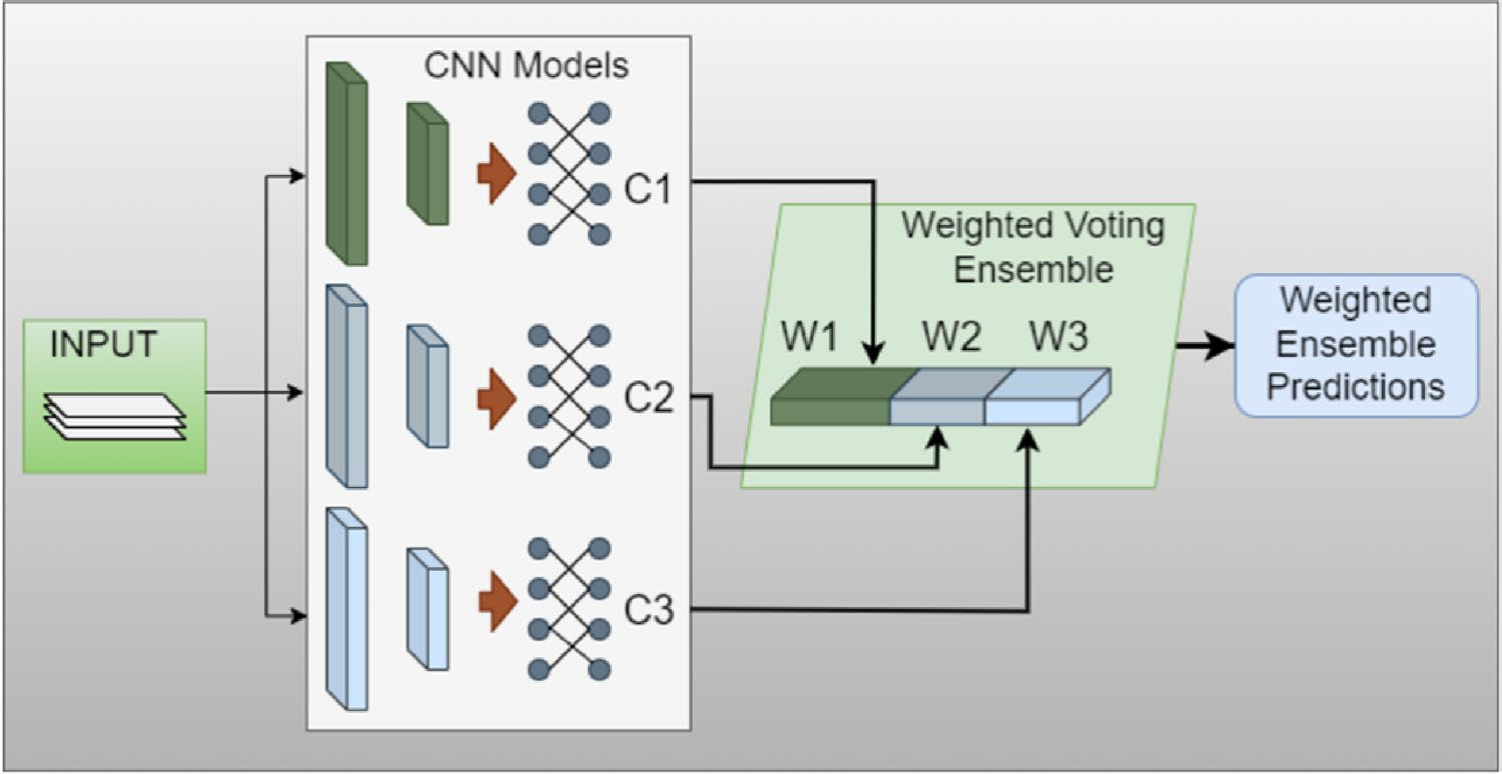
Weighted voting ensemble using convolutional neural networks.

The architecture of the proposed technique using the weighted CNN for blood cancer prediction is depicted in Fig. 2. The relevant details of how it works are provided in the subsequent sections.

#### Weighted voting ensemble

A CNN model’s weighted voting ensemble (W.V.E.) is essential for improving robustness and prediction accuracy^36^. Using ensemble learning techniques is very beneficial in prediction, where accuracy and reliability are critical. This is because the approaches combine the benefits of numerous CNN models, each of which has a unique advantage in collecting different aspects of the data. In addition to improving accuracy, this variety strengthens predictions against noise and outliers by allowing models that are susceptible, by giving lower weights. These ensembles also aid in promoting improved generalization, mitigating overfitting, lowering prediction variance, and balancing biases within individual models. They are a flexible tool for modeling intricate decision limits and adjusting to shifting data patterns because of their versatility and capacity to fine-tune model weights^37^. To sum up, weighted voting ensemble CNN models are a useful tool for achieving more precise and reliable predictions in a variety of applications. In this paper, an ensemble model WV-CNN is proposed which is derived from three 1-D CNN models to attain high accuracy and leverage the robust decision-making strength of multiple models for enhanced cancer prediction. The following lists the model structures of the three contributing CNN models^38^.

#### ECN-1 (ensemble convolutional network 1)

Ensemble convolution network 1 (ECN - 1) is a proposed sequential convolution model fed with a 1-D feature vector v’ of dataset D. According to Table 8, the ECN-1 model is composed of a 1-D convolution layer with a filter number Fn of 4 and a kernel size Ks 3. Except for the last layer, every layer of the model uses ReLU as an activation function. The final layer, which has as many neurons as the length of the classes being trained on with a Softmax Activation to obtain a probability distribution of input labels, comes after the feature extraction layer. This dense layer has six neurons.

#### ECN-2 (ensemble convolutional network 2)

To get class probability values, the second Sequential convolution model, ECN-2, is fed the feature vector v’. Table 8 details the model design, which includes a 1-D convolutional layer with Fn and Ks both set to 3. To prevent over-fitting, a flatten layer, a dense layer with eight neurons, and a dropout layer with a value of 0.3 are added next. With as many neurons as the class length, the final layer is dense.

**Table 8:** CNN ensemble models’ recommended architecture.

Layer	Filters	Neurons	Kernel	Dropout	Activation
ECN-1					
ConvID	4	–	3	–	ReLU
Flatten	–	–	–	–	–
Dense		6	–	–	Relu
Dense	–	Len(classes)	–	–	Sigmoid/softmax
ECN-2					
ConvID	3		3	–	ReLU
Flatten	–		–	–	–
Dense	8		–	–	Relu
Dense	–	Len(classes)	–	–	Sigmoid/softmax
ECN-3					
ConvID	5				ReLU
Flatten	–				–
Dense	–	Len(classes)			Sigmoid/softmax

#### ECN-3 ensemble convolutional network 3

To get classification scores, the proposed Sequential network ECN-2 receives the input feature vector v’, just like in the first two models. The model’s layer structure is represented by a 1-D Convolutional Layer with Fn = 5 and Ks = 3, with ReLU serving as the activation function, as shown in Table 8. The next set of layers uses a softmax classifier and consists of an output-dense layer and a flatten layer with as many neurons as there are classifications that need to be predicted. Table 9 provides the parametric setting for every model in the ensemble. Adam is utilized as the optimizer for all models in the ensemble because of its quicker computing and less complicated parametric configuration requirements. The gradient’s scaling term ($\beta$2) and momentum ($\beta$) are set at 0.999 and 0.9, respectively, while the learning rate ($\alpha$) is set at 0.001. Likewise, a tiny positive number is assigned to the epsilon value to prevent division by zero.

**Table 9:** Configuring parametrically the proposed WVCNN ensemble models.

	ECN-1	ECN-2	ECN-3
Optimizer	Adam	Adam	Adam
L.Rate	0.001	0.001	0.001
Beta 1	0.9	0.9	0.9
Beta 2	0.999	0.999	0.999
Epsilon	1.00E−07	1.00E−07	1.00E−07
Decay	0	0	0
AMS-Grad	False	False	False
Ensemble weight	0.4	0.3	0.3

#### Weighted voting convolutional neural network

The increased prediction values are obtained by an ensemble voting regimen that uses the output probabilities of the three suggested models, ECN1, ECN2, and ECN3. Since each model’s contribution to the final probability depends on a set weight value, these agreed-upon class probabilities represent the outcome of the vote from each model^37,39^. The weights W1, W2, and W3 for each of the suggested models ECN1, ECN2, and ECN3, as shown in Fig. 2, are set at 0.4, 0.3, and 0.3, respectively, just like in our situation. The final probability vectors for the models ECN1, ECN2, and ECN are represented by the symbols p1, p2, and p3 for a given input feature vector V. The mathematical expression for the weighted average of the probability over all three models is given in Eq. (7).

7 $$\begin{aligned} \hat{P}ens=\sum _{i=1}^{n}W_ip_i \end{aligned}$$

Choosing the class with the greatest likelihood is done by Eq. (8)

8 $$\begin{aligned} P_{out}=argmax(\hat{P}ens) \end{aligned}$$

### Evaluation parameter

Performance measures for the machine learning models are evaluated, including recall, precision, F1 score, and accuracy. All these metrics were used in this study for the evaluation of the machine learning models based on the confusion matrix. This matrix is a tabular tool that shows how well the model performs in classifying test data.

#### Accuracy

The accuracy score reflects the precision of a model’s predictions, showing how closely the model’s predictions match the actual results. It serves as a measure that quantifies the model’s capability to make accurate predictions. The accuracy score is computed by dividing the total number of correct guesses by the total number of forecasts. An ideal model achieves a perfect accuracy score of 1, while the lowest possible score is 0. It is defined in Eq. (9)

9 $$\begin{aligned} Accuracy (A)=\frac{TP+TN}{TP+TN+FP+FN} \end{aligned}$$

where,

- True positive (TP): when a patient’s real label is ‘Healthy’ even when the model accurately predicts them to be ‘Healthy’. This shows that the patient’s actual condition and the model’s forecast match.
- True negative (TN): when the model accurately predicts an ‘Un-Healthy’ patient when the actual label corresponds to ‘Un-Healthy’. Similar to TP, TN signifies that the model’s prediction aligns with the patient’s actual condition.
- False positive (FP): when the model incorrectly predicts a patient as ‘Healthy’ while the true label is ’Un-Healthy’. This represents a scenario where the model fails to identify an infected patient, leading to a false sense of normalcy.
- False negative (FN): when the model erroneously predicts a patient as ‘Un-Healthy’ when the true label is ‘Healthy’. FN indicates that the model misclassifies a healthy patient as infected, resulting in unnecessary concern or treatment.

#### Precision

The number of correctly guessed positive cases compared to all the times thought as positive is called precision. The best score a model can get for accuracy is 1. The lowest it can get is 0. Precision is calculated using Eq. (10)

10 $$\begin{aligned} Precision=\frac{TP}{TP+FP} \end{aligned}$$

#### Recall

Recall, also known as True Positive Rate (TPR) or Sensitivity, indicates how successfully the classifier can recognize each and every positive sample. It is calculated by dividing the sum of True Positives (TP) and False Negatives (FN) by the ratio of TP to FN. A model can have a maximum recall score of 1 and a minimum value of 0. Eq. (11) is used to calculate the recall

11 $$\begin{aligned} Recall=\frac{TP}{TP+FN} \end{aligned}$$

#### F1 score

The balance between recall and precision is represented by the F1 score, also known as the F measure. It illustrates how these two metrics might be compromised, with a model obtaining an F1 score of at least 0 and up to 1. F1 score is calculated using Equation 12

12 $$\begin{aligned} F1-Score= 2 \times \frac{Precision\times Recall}{Precision+Recall} \end{aligned}$$

## Experiments and analysis

In this research, we use various machine learning models to identify blood cancer, in addition to the proposed weighted CNN approach. In order to prevent overfitting, the dataset is split into training and testing sets using the standard 85:15 ratio, which is often used in classification studies. We employ a range of metrics intended for machine learning classifiers to assess the models’ performance. The operating system is Microsoft Windows 10 and all experiments are conducted in a Python environment, employing different libraries like sklearn to implement machine learning models, Keras to implement deep learning models, seaborn for visualization, and imblearn for SMOTE sampling. Two$\times$ Intel Xeon 8 Cores processors operating at 2.4 GHz, along with 32 GB of DDR4 RAM, power the Dell PowerEdge T430 GPU, which does the computations.

### Experimental results with the original dataset

Table 10 presents a comprehensive summary of the findings from the initial experiment on machine learning performance using the original blood cancer dataset. In this dataset, the LR, KNN, and SVC models exhibit higher values of accuracy, with the highest accuracy of 91%. Notably, the proposed WVCNN model stands out for its exceptional performance in terms of recall, precision, accuracy, and F1 score, which is 92%, 96%, 90%, and 90% respectively on the original dataset. This shows the significance of the proposed approach in predicting blood cancer. The remarkable performance of WVCNN can be attributed to its hybrid architecture. LR, SVC, and KNN also demonstrate commendable performance, in comparison with the accuracy of WVCNN. On the other hand, ADA is the least performer among other learning models used in this study. It is due to the limited size of the dataset, impeding its boosting approach that relies on a larger number of records for enhanced accuracy. In confusion matrices, B-CELL ALL, B-CELL ALL ETV6-RUNX1, B-CELL ALL HYPERDIP, B-CELL ALL HYPO, B-CELL ALL MLL, B-CELL ALL T-ALL, and B-CELL ALL TCF3-PBX1 are represented by the numbers 0, 1, 2, 3, 4, 5, and 6.

**Table 10:** Learning model result on the original dataset.

Model	Accuracy (%)	Precision (%)	Recall (%)	F1 score (%)
KNN	91	95	88	88
ADA	65	78	67	67
ETC	88	80	84	82
RF	88	81	84	82
NB	86	79	81	79
DT	72	74	72	73
SVC	91	96	88	88
LR	91	95	88	88
MLP	89	93	91	92
ANN	90	94	94	94
WVCNN	92	96	90	90

### Performance of models using SMOTE-Tomek oversampling

The models show better performance after using the SMOTE-Tomek technique, which helps balance the dataset for all classes by generating new data. Enhancing data balance not only enlarges the dataset but also enhances model performance while mitigating the likelihood of overfitting. The outcomes following the implementation of the SMOTE-Tomek technique are outlined in Table 11. LR, RF, SVC, ETC, and WVCNN demonstrate commendable performance, attaining an accuracy score of 97%. Conversely, ADA exhibits subpar performance due to the dataset’s insufficient size, hindering the boosting algorithm’s effective fitting.

**Table 11:** Performance of the models using SMOTE-Tomek oversampling.

Model	Accuracy (%)	Precision (%)	Recall (%)	F1 score (%)
KNN	87	91	88	87
ADA	75	86	78	77
ETC	97	97	97	96
RF	97	97	97	97
DT	87	87	88	87
SVC	97	97	96	97
LR	97	96	97	97
NB	95	95	95	95
MLP	93	94	97	95
ANN	96	94	95	95
WVCNN	97	97	97	97

### Performance of models following implementation of Chi2 technique

The Chi2 method is utilized to evaluate the performance of both the learning models and the proposed system. As indicated by the results in Table 12, Chi2 has a negligible effect on the performance of the models. In particular, WVCNN maintains its original accuracy score of 92%, consistent with the observations from the initial dataset. In the case of the original dataset, the use of Chi2 enhances ADA’s performance from 65% to 72% and DT’s performance from 72 to 74%. It’s important to note that the primary goal is not to surpass SMOTE-TOMEK in terms of model performance. Chi2 serves to decrease complexity and enhance overall performance by choosing only significant features to train the model, resulting in improved performance compared to the original dataset.

**Table 12:** Learning models results with the implementation of the Chi2 technique.

Model	Accuracy (%)	Precision (%)	Recall (%)	F1 score (%)
RF	88	81	83	82
KNN	79	82	81	81
NB	86	85	85	85
ETC	86	79	82	82
DT	74	73	74	73
ADA	72	64	59	59
LR	88	81	83	82
SVC	86	78	82	80
MLP	89	82	86	84
ANN	91	81	84	83
WVCNN	92	83	87	84

### Experimental results integrating Chi2 and SMOTE-Tomek

The models exhibit notably enhanced performance when the preprocessing involves a combination of the SMOTE-TOMEK and Chi2 approaches. By employing SMOTE-TOMEK, new data is generated to mitigate the risk of model overfitting for the majority class, while Chi2 selects the most pertinent features by assessing their correlation with the target class. As depicted in Table 13, the results demonstrate a substantial improvement when both approaches are applied simultaneously. All models perform well on average, but the recommended WVCNN model outperforms the others, achieving a flawless accuracy of 99.9%. In comparison, LR, ETC, and KNN demonstrate accuracy values of 97%, 97%, and 95%, respectively, while RF and SVC attain accuracy scores of 99%.

**Table 13:** Learning models results with the integration of Chi2 and SMOTE-TOMEK.

Model	Accuracy (%)	Precision (%)	Recall (%)	F1 score (%)
RF	99	99	98	98
KNN	95	96	92	92
NB	92	91	90	91
ETC	97	97	96	97
DT	84	87	81	82
ADA	86	88	85	84
LR	97	97	97	97
SVC	99	99	98	98
MLP	98	98	99	98
ANN	98	97	99	98
WVCNN	99	99	99	99

### Experimental results with feature selection after data splitt

This study independently performs feature selection on both the training and testing sets subsequent to data splitting. This approach is adopted to evaluate the significance of WVCNN and to preempt any potential data leakage that might arise if feature selection were to occur prior to the data split. The experimental data for each model is detailed in Table 14, revealing that the suggested model, WVCNN, outperforms all others with the highest accuracy score of 98%. WVCNN distinguishes itself with an accuracy score of 98%, establishing its prominence among all evaluation factors. It is closely followed by LR, RF, and ETC models.

**Table 14:** Learning models results when feature selection after data splitting.

Model	Accuracy (%)	Precision (%)	Recall (%)	F1 score (%)
KNN	89	91	90	89
ETC	96	96	96	96
ADA	38	42	45	39
SVC	95	95	95	95
RF	96	96	96	96
NB	94	94	94	94
DT	86	85	86	85
LR	96	96	96	96
MLP	97	98	96	97
ANN	97	97	97	97
WVCNN	98	98	98	98

Table 15 shows a comparison of the proposed model’s performance involving the original dataset, feature engineering with Chi2, data oversampling using SMOTE-Tomek, and integrating both Chi2 and SMOTE-Tomek. Results show superior performance when Chin2 is used for feature selection on the dataset oversampled using SMOTe-Tomek. These noteworthy findings imply that feature selection can be executed either before or after data splitting without causing data leakage. It is pertinent to highlight that the reported results were attained when feature selection was performed after the data-splitting process.

**Table 15:** Comparison of learning model results employing different feature engineering techniques.

Model	Accuracy (%)	Precision (%)	Recall (%)	F1 score (%)
WVCNN (original)	92	96	90	90
WVCNN with Chi2	92	83	87	84
WVCNN with SMOTE-Tomek	97	97	97	97
WVCNN with Chi2+ SMOTE-Tomek	99	99	99	99.9

### Results using K-fold cross-validation

To ensure the reliability of the models, we employed a technique known as K-fold cross-validation. The outcomes of this five-fold cross-validation are detailed in Table 16. The table distinctly illustrates that our proposed approach outperforms other models in terms of accuracy, precision, recall, and F1 score, demonstrating minimal variability. This suggests that our approach is not only effective but also consistently performs well.

**Table 16:** 5-fold cross-validation results for the proposed approach.

Model	Accuracy	Precision	Recall	F1 score
1st fold	99.25	99.31	99.16	99.21
2nd fold	99.25	99.43	99.47	99.32
3rd fold	99.46	99.76	99.89	99.18
4th fold	99.80	99.87	99.76	99.85
5th fold	99.89	99.51	99.68	99.49
Average	99.61	99.72	99.54	99.41

#### Performance validation of weighted CNN

In this section, we have done performance validation of the proposed model utilizing two scenarios. In the first scenario, we utilized another independent 2023 cancer dataset and tested our model performance on it^40^. This dataset includes patient attributes for those diagnosed with cancer. It features a unique identifier for each patient, the type of cancer (diagnosis), and the average values of these characteristics. The dataset also includes several categorical features where patients are assigned numerical values for attributes such as texture_mean, radius_mean, perimeter_mean, smoothness_mean, area_mean, compactness_mean, concave points_mean, and concavity_mean. Results reveal that the proposed model gives 98.94% accuracy, 98.98% precision, 99.25% recall, and 99.15% F1 score. These results values show the superiority and stability of the proposed model on a diverse dataset.

In the second scenario, we trained our proposed model on chi2 + SMOTE-Tomek significant features and tested the proposed model on original dataset features. The results reveal that the proposed model performs quite well if we compare the results of Table 8 in which the model trained and tested on original features of the dataset. The proposed model gives 96.52% accuracy, 98.15% precision, 96.37% recall, and 97.67% F1 score.

#### Performance of weighted CNN concerning existing studies

The suggested approach’s performance is compared with several cutting-edge methods in order to confirm its efficacy and superiority; the outcomes are shown in Table 17. These papers were chosen because their experiments were conducted using the same dataset. For example, Castillo et al.^15^ examined a range of machine learning models, with RF achieving an accuracy of 97.28 percent. Similar to this, Nazari et al.^14^ predicted blood cancer with 96.6 percent accuracy by using a deep learning technique. Our method produced 100% accuracy on the same dataset when compared to this research, indicating the importance of the suggested method.

**Table 17:** Proposed approach comparison with other state-of-the-art systems.

References	Year	Model	Dataset	Accuracy
^15^	2019	NB, RF, KNN, and SVM	Microarray gene	NB—97.29%, KNN—96.28%, RF—97.01
^14^	2020	Deep learning networks, or DNNs	Microarray gene	96.60%
^13^	2022	LDSVM	Microarray gene	99.8%
This study	2024	WVCNN	Microarray gene	99.9%

## Conclusion

This study’s main goal is to use an unbalanced dataset to predict the leukemia type of blood cancer. Accuracy with imbalanced and high-dimensional datasets is still a constant and difficult quest despite the abundance of known methods. A hybrid weighted CNN learning model is presented as a response to this. This model predicts cancer by using leukemia microarray gene data. The SMOTE-Tomek oversampling technique is utilized due to the dataset’s imbalance, and Chi-square is employed to remove characteristics that show minimal association with the target class, hence mitigating the high dimensionality problem. Extensive experiments are carried out with the proposed hybrid approach that incorporates both methodologies and with the SMOTE-Tomek and Chi-square approaches independently. SMOTE-Tomek helps improve the performance of the proposed weighted CNN model as well as machine learning models. By lowering the risk of model overfitting, the oversampling method helps to mitigate class imbalances and improve performance. On the other hand, Chi-square only little affects prediction accuracy. Although Chi-square reduces complexity by training models with only the most pertinent features, not all models benefit from improved performance. Weighted CNN performs better in the suggested method, which combines the use of SMOTE-Tomek and Chi-square, and achieves an impressive 99.9% accuracy in cancer prediction. Utilizing the optimized features, the hybrid architecture of weighted CNN achieves a satisfactory fit and generates predictions based on a majority voting criterion. Furthermore, a comparison with cutting-edge methods confirms the legitimacy and superiority of the proposed method. The results imply that an ensemble of models can perform better than a single model. The creation of a customized deep learning model that is suited for small datasets is planned for future endeavors. Additionally, integrating several datasets to produce a complex and high-dimensional dataset is being considered for use in studies utilizing the proposed approach.

## Data Availability

This study used a publicly available Leukemia_GSE28497 dataset which can be accessed using the following link: https://www.ncbi.nlm.nih.gov/geo/query/acc.cgi?acc=GSE28497.

## References

[1] Talukder MA (2023). An efficient deep learning model to categorize brain tumor using reconstruction and fine-tuning. Expert Syst. Appl..

[2] Talukder MA (2022). Machine learning-based lung and colon cancer detection using deep feature extraction and ensemble learning. Expert Syst. Appl..

[3] Sharmin S, Ahammad T, Talukder MA, Ghose P (2023). A hybrid dependable deep feature extraction and ensemble-based machine learning approach for breast cancer detection. IEEE Access.

[4] Centre, W. H. O. M. *Cancer Fact Sheet* (2020).

[5] Horng JT (2009). An expert system to classify microarray gene expression data using gene selection by decision tree. Expert Syst. Appl..

[6] Rupapara V (2022). Blood cancer prediction using leukemia microarray gene data and hybrid logistic vector trees model. Sci. Rep..

[7] Castillo D (2019). Leukemia multiclass assessment and classification from microarray and RNA-seq technologies integration at gene expression level. PloS one.

[8] Veeraiah N, Alotaibi Y, Subahi AF (2023). Maygan: Mayfly optimization with generative adversarial network-based deep learning method to classify leukemia form blood smear images. Comput. Syst. Eng..

[9] Ideker T, Thorsson V, Siegel AF, Hood LE (2004). Testing for differentially-expressed genes by maximum-likelihood analysis of microarray data. J. Comput. Biol..

[10] Nekoeian S (2023). Identification of lncrnas associated with the progression of acute lymphoblastic leukemia using a competing endogenous rnas network. Oncol. Res..

[11] Veeraiah N, Alotaibi Y, Subahi AF (2023). Histogram-based decision support system for extraction and classification of leukemia in blood smear images. Comput. Syst. Eng..

[12] Gupta S, Gupta MK, Shabaz M, Sharma A (2022). Deep learning techniques for cancer classification using microarray gene expression data. Front. Physiol..

[13] Karim A, Azhari A, Shahroz M, Belhaouri SB, Mustofa K (2021). Ldsvm: Leukemia cancer classification using machine learning. Comput. Mater. Sci..

[14] Nazari, E. *et al.* Deep learning for acute myeloid leukemia diagnosis. *J. Med. Life* . 10.25122/jml-2019-0036 (2020). 10.25122/jml-2019-0090 PMC7550141 33072212

[15] Castillo D (2019). Leukemia multiclass assessment and classification from microarray and RNA-seq technologies integration at gene expression level. PloS one.

[16] Fauzi IR, Rustam Z, Wibowo A (2021). Multiclass classification of leukemia cancer data using fuzzy support vector machine (FSVM) with feature selection using principal component analysis (pca). J. Phys. Conf. Ser..

[17] Abd El-Nasser, A., Shaheen, M. & El-Deeb, H. Enhanced leukemia cancer classifier algorithm. In *2014 Science and Information Conference*. 422–429. 10.1109/SAI.2014.6918262 (IEEE, 2014).

[18] Mehrabani, S., Soroush, M. Z., Kheiri, N., Sheikhpour, R. & Bahrami, M. Prediction of blood cancer using leukemia gene expression data and sparsity-based gene selection methods. *Iran. J. Pediatric Hematol. Oncol.*10.18502/ijpho.v12i3.5753 (2022).

[19] Mahdi, G. J., Kalaf, B. A. & Khaleel, M. A. Enhanced supervised principal component analysis for cancer classification. *Iraqi J. Sci.* **62**, 1321–1333 10.24996/ijs.2021.62.4.6 (2021).

[20] Loey M, Naman M, Zayed H (2020). Deep transfer learning in diagnosing leukemia in blood cells. Computers.

[21] Vijayarani, S. & Sudha, S. An efficient clustering algorithm for predicting diseases from hemogram blood test samples. *Indian J. Sci. Technol.*10.17485/ijst/2015/v8i1/60103 (2015).

[22] Ancona N (2006). On the statistical assessment of classifiers using DNA microarray data. BMC Bioinform..

[23] Song, G. *New Markers for Minimal Residual Disease Detection in Acute Lymphoblastic Leukemia*. https://www.ncbi.nlm.nih.gov/geo/query/acc.cgi?acc=GSE28497. Accessed 3 Aug 2023 (2018). 10.1182/blood-2010-12-324004 PMC3122946 21487112

[24] Hameed, A. *et al.* Skin lesion classification in dermoscopic images using stacked convolutional neural network. *J. Ambient Intell. Hum. Comput.* 1–15 (2021).

[25] Ijaz MF, Attique M, Son Y (2020). Data-driven cervical cancer prediction model with outlier detection and over-sampling methods. Sensors.

[26] Abdoh SF, Rizka MA, Maghraby FA (2018). Cervical cancer diagnosis using random forest classifier with smote and feature reduction techniques. IEEE Access.

[27] Kleinbaum, D. G., Dietz, K., Gail, M., Klein, M. & Klein, M. *Logistic Regression* (Springer, 2002).

[28] Sarwat S (2022). Predicting students’ academic performance with conditional generative adversarial network and deep svm. Sensors.

[29] Juna A (2022). Water quality prediction using knn imputer and multilayer perceptron. Water.

[30] Juna A (2022). Water quality prediction using knn imputer and multilayer perceptron. Water.

[31] Rish, I. *et al.* An empirical study of the Naive Bayes classifier. In *IJCAI 2001 Workshop on Empirical Methods in Artificial Intelligence*. Vol. 3. 41–46 (2001).

[32] Geurts P, Ernst D, Wehenkel L (2006). Extremely randomized trees. Mach. Learn..

[33] Kotsiantis SB (2013). Decision trees: A recent overview. Artif. Intell. Rev..

[34] Friedman, J. H. Greedy function approximation: a gradient boosting machine. *Ann. Stat.* 1189–1232 (2001).

[35] Yegnanarayana, B. *Artificial Neural Networks* (PHI Learning Pvt. Ltd., 2009).

[36] Hafeez, U. *et al.* A CNN based coronavirus disease prediction system for chest X-rays. *J. Ambient Intell. Hum. Comput.* 1–15 (2022). 10.1007/s12652-022-03775-3 PMC8882219 35251361

[37] Umer M (2022). IoT based smart monitoring of patients’ with acute heart failure. Sensors.

[38] Ahmad M (2022). Industry 4.0 technologies and their applications in fighting covid-19 pandemic using deep learning techniques. Comput. Biol. Med..

[39] Cascone L (2023). Predicting household electric power consumption using multi-step time series with convolutional LSTM. Big Data Res..

[40] Taha, E. *Cancer Data*. https://www.kaggle.com/datasets/erdemtaha/cancer-data. Accessed 15 May 2024 (2023).

